# Selections

**Published:** 1870-10

**Authors:** 


### Selections.

Caoutchouc, or India rubber, as it is more commonly termed, is obtained from the milky juice of trees and vines of different countries. It is exported in its crude state from South America, Central America, Mexico, East Indies, and Africa; the rubber obtained from these countries differing materially from each other, which is due, to some extent, to the different methods adopted in curing it.

The best and leading quality is considered to come from Para, Brazil, S. A., termed "Para Rubber." It is gathered by the natives in the interior of Brazil, from trees of large growth, which are "tapped" once a year, by making incisions in the bark, commencing near the base of the tree, some times in the roots which lie exposed. These incisions are repeated once a fortnight during the season for gathering rubber, continuing them up the trunk of the tree. It is stated that the production from the roots which lie exposed is more abundant in the elastic gum, than any which is subsequently drawn off. A milky emulsion containing the caoutchouch exudes at these orifices, which is collected by means of gourds or cocoa nuts attached to the tree by means of clay; these, when full, are emptied into buckets. A fire is then made from nuts peculiar to this country, which in burning emit a dense smoke that has the property of curing and drying the rubber in a superior manner. Molds of a variety of forms, but principally made from wood about one inch in thickness, either round or oval, are then dipped into the liquid and passed through the smoke; when the gum becomes dry and solid they are again applied to the liquid for a second coating, and redried; this process being continued until the coating becomes from half to one inch in thickness; the layer is then cut open, the mold removed, and the rubber is ready for shipment to Para, from which port it is shipped to the markets of consumption. The finest and best of this production is termed "Fine Para;" that which is a little inferior in quality or texture, "secon," or "medium;" while all the scraps and refuse are compressed into large balls or blocks, and called "surnamby," "coarse," or "negro-head." Much has been said relative to the exhaustion of this supply. The trees, if not abused, will yield for an almost indefinite period, the older trees furnishing a juice which is much richer in caoutchouc than any others. The increased production of this rubber is readily shown by the following table of exports from Para:

| | To United States. | To Europe. |
|---|---|---|
| 1851 | 1,700,000 lbs. | 1,250,000 lbs. |
| 1855 | 2,650,000 " | 2,150,000 " |
| 1860 | 2,300,000 " | 2,800,000 " |
| 1865 | 3,000,000 " | 5,100,000 " |
| 1869 | 5,700,000 " | 4,850,000 " |

Rubber from New Granada, Ecuador, and Central America, is exported in a variety of forms, namely, slabs, sheets and pressed strip. This rubber is cured in an entirely different manner from the Para rubber; the milk after it is collected being allowed to coagulate, and then run into thin sheets or slabs, the latter varying from one to four inches in thickness, which are dried as thoroughly as possible in the sun. The well rubber so cured contains much acid natural to the milk, as a large percentage of water. The pressed strip rubber is all exported from Panama, where it is collected from the interior in slabs, which are cut into narrow strips and run through powerful presses, extracting the water, compressing the rubber, and preparing it for the market in a better condition than when shipped in the wet state. Mexican, East India and African rubber are all cured in the same manner as Central American rubber. In Africa, most of the rubber-milk is collected from a large vine, similar to our native grape vine. Although the trees grow there in abundance, it is all collected in the district surrounding Gaboon, directly on the equator. The rubber trees are very numerous on the gold coast in Guinea further north, but there is no rubber collected in that district. Much of the African rubber is prepared by filling reeds from six to ten inches in length with milk from the vine, which, when hardened, is taken out and left in the sun to dry. So prepared it is termed tongue. The other quality, flake, comes in different shapes and sizes, but is not so free from dirt, being allowed to run upon the ground and dry, after which it is collected. The best African rubber comes from the Congo river, about six degrees south of the equator. It is prepared in the shape of balls. and termed ball rubber. The East India and Africa rubber is of a softer and more gummy nature than the other kinds of rubber, and is generally used in manufacture for a sticking substance, and in the manufacture of hose. The annual consumption of all kinds of rubber in the United States in 1858 amounted to about 2,500,000 pounds, which has now increased to from 9,000,000 to 10,000,000 pounds.

MENTAL STRAIN AS A DISEASE.—Dr. B. W. Richardson, of London, has recently referred to the importance of studying the influence of impressions made through the mind upon the organs of the body.

He contends that when the mental powers are so greatly overtasked, as they are at the present time, this study claims the immediate attention of the general as well as the psychological physician. Dr. Richardson, in describing the different classes of men who are engaged in labors which call forth the energies of the mind, divides them into "copyists, authors, artists, speculators, professionals and students. He defines the nature of the work of these respectively, and the diseases to which they are subject as the effect of their labors.

Turning to the diseases in detail, Dr. Richardson places not only paralysis, insanity, epilepsy, intermittent action of the heart, and arterial enervation with symptoms simulating aneurism, but also diabetes and cancer, as diseases directly connected with mental strain in persons who have an hereditary tendency to them.

As relates to insanity itself, the author holds an opinion different to that commonly entertained, in his asserting that work, and even hard work of a mental character, does not of itself bring on the disease, but that by such work the organs of the body which are purely physical are most severely injured. Insanity is, he thinks, a disease growing out of ignorance and mental inactivity; so that, in fact, the sluggish parts of the population are the most determinate producers of insanity, and the educated and most intelligent parts of our population are the producers and propagators of the more serious and fatal of organic diseases.

But in reasoning on the share which undue strain on the brain—the material organ of the mind—has in causing insanity, there is one important distinction to be constantly remembered, viz: that between the faculties purely intellectual on the one side, and the feeling, emotions and passions, which make up the largest portion of the functions of the brain, on the other. More commonly it will be found that insanity is the result of excessive indulgence and contentions in these latter, rather than in a simply severe taxing of the intellect alone.

This belief will be fully borne out by the history of genius in all its varied manifestations. And again, the intellect of a student is often supposed to have become weakened, if not lost, by too close and continuous labor, when, in fact, the result was chiefly due to irregular hours, strong drinks, and not seldom tobacco or opium."

